# Neuropsychological outcome of indoor rehabilitation in post-COVID-19 condition—results of the PoCoRe study

**DOI:** 10.3389/fneur.2024.1486751

**Published:** 2025-01-06

**Authors:** Michael Jöbges, Melanie Tempfli, Christoph Kohl, Christoph Herrmann, Stefan Kelm, Alexa Kupferschmitt, Ida Montanari, Nike Walter, Gerhard Suetfels, Thomas Loew, Volker Köllner, Thilo Hinterberger

**Affiliations:** ^1^Kliniken Schmieder Konstanz, Konstanz, Germany; ^2^Department of Psychology, University of Konstanz, Konstanz, Germany; ^3^Westerwald Clinic Waldbreitbach, Waldbreitbach, Germany; ^4^Kliniken Schmieder Gailingen, Gailingen, Germany; ^5^Research Group Psychosomatic Rehabilitation, Department of Psychosomatic Medicine, Charité Universitätsmedizin, Berlin, Germany; ^6^Rehabilitation Clinic Seehof, Department of Psychosomatic Medicine, Federal German Pension Agency, Teltow, Germany; ^7^Department of Psychosomatic Medicine, University Hospital Regensburg, Regensburg, Germany; ^8^Todtmoos Rehabilitation Centre, Wehrawald Clinic, Federal German Pension Agency, Todtmoos, Germany

**Keywords:** post COVID condition (PCC), neurologic rehabilitation, neuropsychology, fatigue, Montreal Cognitive Assessment (MoCA)

## Abstract

**Background:**

Post COVID-19 condition (PCC) is increasingly recognized as a debilitating condition characterized by persistent symptoms following SARS-CoV-2 infection. Neuropsychological deficits, including cognitive impairments and fatigue, are prevalent in individuals with PCC. The PoCoRe study aimed to evaluate the burden of neuropsychological deficits in PCC patients undergoing multidisciplinary indoor rehabilitation and to describe possible changes in this symptomatology.

**Methods:**

The PoCoRe study, a prospective, non-randomized, controlled longitudinal study, recruited PCC patients from six German indoor rehabilitation centers. Eligible participants underwent comprehensive neuropsychological assessments at admission and discharge. Various measures were employed, including the fatigue scale for motor functioning and cognition (FSMC), the Test Battery for Attention (TAP) and the Montreal Cognitive Assessment (MoCA).

**Results:**

Out of the 1,086 recruited participants, a total of *N* = 701 participants were included in the main data analysis. The prevalence of fatigue on admission was high (84.6%) and decreased significantly by discharge (77.4%), with a mild effect size. Reaction times on the alertness subtest were abnormal in 70% of patients on admission and 50% on discharge. Sustained attention was abnormal in 55% of patients on admission, decreasing to 43% on discharge. These differences were significant with mild effect sizes. Furthermore, of the 27% of participants with pathological MoCA scores at admission, 63% improved to normative levels during rehabilitation, indicating a significant treatment effect (*p* ≤ 0.001). However, the MoCA demonstrated limited sensitivity in detecting attention deficits.

**Conclusion:**

The PoCoRe study highlights the high prevalence of neuropsychological deficits and fatigue in PCC patients, with notable improvements observed following multidisciplinary rehabilitation. Challenges remain in accurately identifying and addressing these deficits, underscoring the importance of comprehensive neuropsychological assessment and tailored rehabilitation interventions. Further research is warranted to optimize screening tools and enhance neuropsychological care for PCC patients in both rehabilitation and outpatient settings.

## Introduction

The COVID-19 pandemic, caused by the novel coronavirus SARS-CoV-2, has left an indelible mark on global health, society, and economies. While much attention has rightfully focused on acute illness and preventive measures, the aftermath of the virus is increasingly coming to light. Among the emerging concerns is the phenomenon known as Post COVID-19 condition (PCC).

PCC occurs in individuals with a history of probable or confirmed SARS-CoV-2 infection, usually 3 months from the onset, with symptoms that last for at least 2 months and cannot be explained by an alternative diagnosis (1).

At least 65 million people worldwide are estimated to have PCC (2).

The most prevalent PCC symptoms two-years after SARS-CoV-2 infection are fatigue and cognitive impairments (3–5). In a comprehensive neuropsychological evaluation patients with PCC showed significantly lower scores in domains of memory, language, processing speed, visuospatial function, executive function, and higher depressive and anxiety symptoms (6).

Recognizing the urgent need for targeted interventions to address the complex sequelae of PCC, the PoCoRe study was initiated as a collaborative effort across six German indoor rehabilitation centers specializing in neurologic or psychosomatic care. The centers adopted their therapeutic concept to the specific needs of the PCC patients (7).

The present study aims to elucidate the symptom burden of neuropsychological deficits in individuals grappling with PCC while also evaluating the efficacy of a multidisciplinary indoor rehabilitation program in ameliorating these challenges. By systematically assessing cognitive function, and functional outcomes before and after rehabilitation, this research aims to contribute valuable insights into the long-term management of PCC and inform the development of targeted interventions to support affected individuals on their path to recovery.

## Methods

The PoCoRe study is a prospective, non-randomized, controlled longitudinal study in Germany. A study protocol has been published previously (7).

The study took place in six indoor rehabilitation clinics specialized in neurological rehab and psychosomatic rehab. It was funded by the German pension found (Deutsche Rentenversicherung), so insured persons could take part from March 2022 to December 2023. All consecutively admitted rehabilitation patients who started rehabilitation as a result of PCC and who met the eligibility criteria as well as consent to participate in the study were included.

### Eligibility criteria

- SARS-CoV-2-infection and following PCC: Complaints that are still present more than 12 weeks after the onset of SARS-CoV-2 infection and cannot be explained otherwise.
- As a consequence of PCC at the time of the start of rehabilitation the presence of functional limitations that may threaten the ability to work.
- Written informed consent to participate in the study.
- Aged 18 years or above.
- Sufficient knowledge of the German language to participate in the study.
- Patients with ME/CFS were not excluded. However, we did not assess ME/CFS symptoms systematically within our study design.

### Interventions

The clinics and their treatment programs are reported previously (7). The specializations diverge in the duration and frequency of the therapies, but uniformly cover all essential symptom areas of the PCC with corresponding offers (psychoeducation, exercise, psychotherapy, pacing, breathing and relaxation therapy, cognitive training). Within in this framework, the treatment program was adapted to the individual needs of the patient, including adaptations for individuals affected by ME/CFS (see Supplementary Table 1 for comprehensive information).

### Study variables

Trained psychologists conducted the study-related examinations/tests on admission to rehabilitation and on discharge. The participants completed the questionnaires autonomously (for a full listing of the questionnaires used, see the study protocol) (7).

### Measures/outcomes

#### Neuropsychology

##### Montreal cognitive assessment

The MoCa is a cognitive screening instrument. It is performed by healthcare professionals to detect early stages of dementia and mild cognitive impairment. It includes subtests for various cognitive abilities such as memory, language, contextual thinking, attention and concentration, behavior, arithmetic, temporal and spatial orientation and the ability to recognize complex shapes and patterns, while also taking into account educational background by awarding an extra point if they have not completed at least 12 years of education. Scores of 26 to 30 points are considered unremarkable, no limitations, 6 to 25 points indicate at least mild cognitive impairment, and 0 to 5 points are interpreted as extreme mental impairment (8).

##### TAP-test

The Test Battery for Attention (TAP) is a software package that offers a collection of different tests that can be used to measure the various aspects of attention in a differentiated way and also cover related aspects of visual perception. Used subtests are alertness, working memory, sustained attention, and divided attention. The battery we chose took about 45 min to complete (9) (see Table 1). Detailed information on the TAP is openly available openly available at https://www.psytest.net/en/test-batteries/tap/subtests.

**Table 1:** Overview of the TAP subtests used.

Subtest	Cognitive domain	Information about
Alertness	Intensity of attention	Basal reactivity, general processing speed, reaction stability, distinction tonic alertness
Working memory	Executive functions, control of the focus of attention	Ability to continuously update the content of working memory
Sustained attention	Intensity of attention	Longer-term maintenance of attention with high target stimulus density
Divided attention	Attentional selectivity, focused attention, visuo-spatial attention	Ability to focus attention on two tasks simultaneously

#### Assessment of fatigue

##### Fatigue scale for motor functioning and cognition (FSMC)

The FSMC is a self-rating scale for assessment of subjective symptoms of fatigue and provides a differential quantification and grading of cognitive and motor fatigue. The FSMC was tested against several external criteria (e.g., cognition, motivation, personality and other fatigue scales) and provides satisfactory results with regard to the test quality criteria. Twenty items (10 for motor fatigue, 10 for cognitive fatigue) are rated using a five-point scale (strongly disagree to strongly agree) (10).

#### Data management and analysis

The above outcomes were assessed by patient questionnaires (FSMC, MoCA) and cognitive assessment (TAP) or extracted from rehabilitation discharge letters (e.g., primary and demographic data; socio-medical data). For more information on the full assessment included in the PoCoRe study, we have uploaded in Supplementary Table 2. If participants withdrew informed consent, the collected data was deleted. The patient data were stored anonymously in an electronic study-data file with a patient cipher, whereby the paper–pencil data were entered in an automated conversion procedure and partly manually. Paper-pencil questionnaires were stored in locked filing cabinets and electronic data were stored on secure servers. To ensure a safe and secure environment for the data collected, data transmission was encrypted using Secure Socket Layer (SSL) technology.

#### Statistical methods

Statistical analyses was conducted using R version 4.3.0 (9), including the stats package, and ggplot2 package (11). Descriptive analyses were performed concerning FSMC, TAP tests, MoCA and sociodemographic data. Indoor rehabilitation effects were calculated employing the Wilcoxon signed-rank test for FSMC and ANOVA for TAP test and MoCA. *p*-values were set to <0.05 and adjusted using the Bonferroni-Holmes method. For Wilcoxon Signed Rank Tests, the effect size r was calculated using the following formula: *r* = z/sqrt(N) (12).

In addition, sensitivity and specificity of the MoCA predicting results of the alertness testing was evaluated (13).

#### Ethics and study registration

All participants provided written informed consent in accordance with the Declaration of Helsinki. The study was approved by the responsible ethics committees [reference numbers: University Hospital Regensburg (including Berlin and Gelderland Klinik): Z-2022-1749-8; Westerwaldklinik: Landesärztekammer Rheinland Pfalz: 2022–16,395; Konstanz and Gailingen: University of Constance 25/2022; Todtmoos: Landesärtzekammer Baden-Württemberg: B-F-2022-032]. The PoCoRe study was registered 03 February 2022 at https://studienanmeldung.zks-regensburg.de.

## Results

Out of the 1,086 recruited participants, a total of *N* = 701 participants were included into data analysis, based on having complete data in the alertness subtest of the TAP (admission and discharge). They had a mean age of 49.5 years (range: 21 to 65, SD = 10.10) and 70.9% were female. The initial infection occurred on average 29 months ago (range: 9 to 50, SD = 8.11).

There was no significant difference concerning age, FSMC values and TAP values on admission between the completed sample and the dropouts at discharge. Because of violated assumptions for parametric testing, the non-parametric Mann–Whitney U test was used to compare MoCA scores at admission. There was a statistically significant difference (U = 79913.00, *p* = 0.003) in MoCA scores between the completed sample (M = 26.53, SD = 2.61) and the dropout sample (M = 25.82, SD = 3.54) with a small effect size of r = 0.10. Within the complete sample, 177 out of 657 individuals (27%) showed a MoCA score below cut-off (<26). This proportion is significantly different from the 105 out of 277 individuals (38%) within the dropouts (Chi-squared = 10.602, df = 1, *p* = 0.0001).

Prevalence of Fatigue on admission was high, to discharge it decreased significantly with a mild effect size (see Table 2).

**Table 2:** Prevalence of deficits and Wilcoxon signed rank test comparing TAP and fatigue measures at admission (T1) and discharge (T2).

	Prevalence of deficits (*n_def_*)		Mean (SD)		Median		*n*	*z*	*p*	*p_adj_*	*r*
	T1	T2	T1	T2	T1	T2					
TAP measures											
Alertness	70.2% (492)	49.5% (347)	33.80 (10.38)	38.41 (11.23)	34	40	701	−13.13	< 0.001	< 0.001	**−0.50**
Working memory	25.6% (176)	19.5% (134)	45.68 (8.12)	47.45 (8.01)	48	48	686	−5.71	< 0.001	< 0.001	−0.22
Sustained attention	55.5% (371)	43.0% (291)	39.27 (10.89)	42.22 (11.44)	39	42	676	−7.96	< 0.001	< 0.001	**−0.31**
Divided attention	33.2% (224)	25.8% (171)	45.27 (12.44)	47.70 (12.47)	46	52	662	−5.27	< 0.001	< 0.001	−0.20
Deficit-subgroups TAP											
Alertness_def_			28.42 (6.60)	34.96 (10.66)	29	36	492	−14.02	< 0.001	< 0.001	**−0.63**
Working memory_def_			35.04 (2.84)	41.27 (7.52)	35	40	172	−9.22	< 0.001	< 0.001	**−0.70**
Sustained attention_def_			31.35 (6.37)	36.51 (10.30)	34	37	358	−9.88	< 0.001	< 0.001	**−0.52**
Divided attention_def_			30.80 (5.36)	38.69 (12.59)	31	37	213	−8.54	< 0.001	< 0.001	**−0.59**
Fatigue (FSMC)											
FSMC_total	84.6% (550)	77.4% (501)	76.86 (15.13)	73.56 (17.62)	80	77	647	−7.50	< 0.001	< 0.001	−0.29
FSMC_cognitive	77.7% (505)	71.7% (464)	38.68 (8.28)	37.08 (9.35)	40	39	647	−6.56	< 0.001	< 0.001	−0.26
FSMC_motor	83.4% (542)	77.0% (498)	38.18 (7.60)	36.48 (8.85)	39	38	647	−7.35	< 0.001	< 0.001	−0.29

TAP measures: Age-, gender-, and education-corrected T-scores at admission (T1) und discharge (T2). Cut-off value for deficits: T < 40. Alertness: Median of reaction times without warning signal. Working memory: number of omissions. Sustained attention: number of omissions. Devided attention: number of omissions. Deficit-subgroups TAP: We performed Wilcoxon-Signed-Rank-Tests for the subgroups containing the participants who showed deficits at T1 in the corresdponding TAP measure. Fatigue (FSMC): Sum scores are displayed; cut-off values for deficits correspond to severe fatigue: FSMC_total (>62), FSMC_cognitive (>33); FSMC_motor (>31). *p_adj_* = *p*-values corrected for multiple testing, using Bonferroni-Holmes correction. *r* = effect size r (32), medium effect sizes are highlighted in bold, large effect sizes bold and underlined.

The results of the TAP subtests were converted to T values. T values lower than 40 were considered to be abnormal (14).

**Figure 1:**
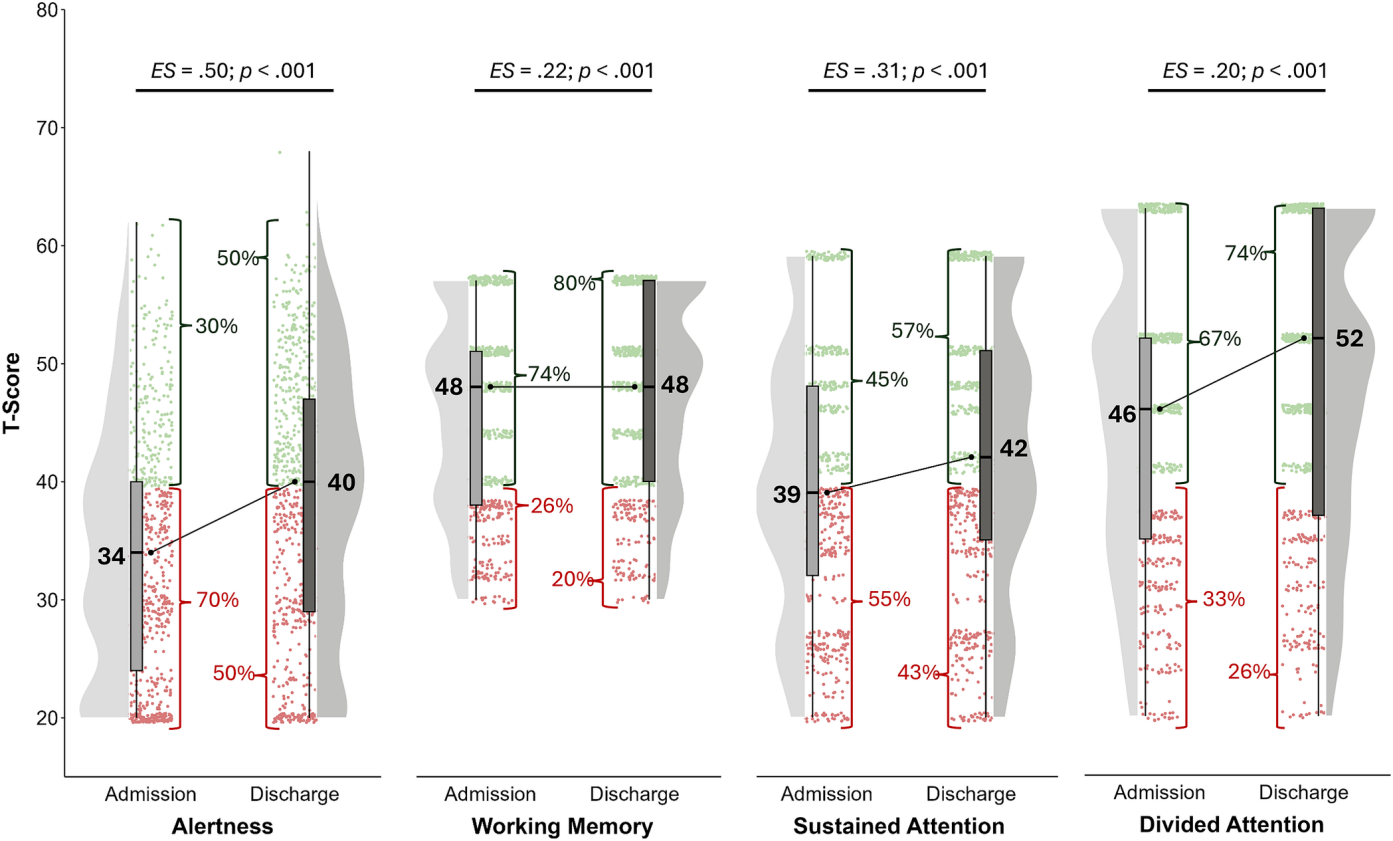
Age-, gender-, and education-corrected T-scores at admission (T1) und discharge (T2). Values in red represent impaired performance (T < 40), while green values are interpreted as at least average. Alertness: Median of reaction times without warning signal; *n_T1_* = 701; *n_T2_* = 701. Working memory: number of omissions; *n_T1_* = 688; *n_T2_* = 686. Sustained attention: number of omissions; *n_T1_* = 681; *n_T2_* = 676. Divided attention: number of omissions; *n_T1_* = 674; *n_T2_* = 662.

The reaction times of the alertness subtest scored abnormal in 70% of patients on admission and 50% on discharge (see Figure 1).

Sustained attention (depicted as the number of omitted answers) was abnormal in 55% on admission with a decrease to 43% on discharge. These differences were significant with mild effect sizes (see Figure 1).

Working memory was impaired in 26% at baseline and 20% at discharge. A significant and mild effect (see Figure 1).

Divided attention scored abnormal in 33% on admission with a decrease to 26% on discharge: a significant and small effect, too (see Figure 1).

The treatment effects for sustained attention, working memory and divided attention were even larger when we took only those participants into account scoring with abnormal results on admission with a T < 40 (see Table 2).

To evaluate the clinical relevance of the observed group-level improvements, we additionally calculated the proportion of individuals who improved on a clinically relevant level, according to critical differences in T-values (T_diff_) provided within the manual of the TAP (14).

At discharge, 37 participants (5.4%) showed clinically relevant improvements in working memory compared to admission (T_diff_ ≥ 14.079), while 10 participants (1.5%) showed clinically relevant worsening. In divided attention, 98 participants (15.1%) showed clinically relevant improvements (T_diff_ ≥ 13.060), while 32 participants (4.9%) showed clinically relevant worsening. In sustained attention, 119 participants (17.9%) showed clinically relevant improvements (T_diff_ ≥ 10.312), while 52 participants (7.8%) showed clinically relevant worsening. At discharge, 341 participants (48.6%) showed clinically relevant improvements in alertness compared to admission (T_diff_ ≥ 3.791), while 82 participants (11.7%) showed clinically relevant reductions in reaction times.

Improvements in alertness being the most prevalent finding on both the group and individual level, we performed further post-hoc analysis evaluating whether the patients significantly improving in alertness also significantly improve in fatigue measured by the difference in FSMC scores. As indicated by *Wilcoxon Signed Rank Tests*, patients who showed clinically relevant improvements in alertness showed higher improvements in overall fatigue and cognitive fatigue with small effect sizes, but no significant improvements in motor fatigue (see Table 3).

**Table 3:** Wilcoxon signed rank test comparing changes in fatigue scores (T1-T2) for patients clinically improving (A_improve_) and not improving (A_non-improve_) in alertness.

	Mean (SD)		Median (*n*)		*z*	*p*	*p_adj_*	*r*
	*A_improve_*	*A_non-improve_*	*A_improve_*	*A_non-improve_*				
Changes in fatigue (FSMC)								
FSMC_total_change	3.84 (13.76)	2.57 (9–31)	2.5	2.0	2.33	0.020	0.040	0.09
FSMC_cognitive_change	1.93 (7.24)	1.15 (5.09)	1.5	1.0	2.80	0.005	0.015	0.11
FSMC_motor_change	1.92 (7.12)	1.42 (4.81)	1.0	1.0	1.15	0.249	0.249	0.04

Patients with clinically relevant improvement (A_improve_; n_improve_ = 341) are identified by increases in alertness reaction times at discharge compared to admission (median without warning signal) by at least *T* = 3.791; patients with lower differences are part of the non-improvement group (A_non-improve_; n_non-improve_ = 360). Changes in Fatigue Scores are measured by the difference in the FSMC at admission (T1) and discharge (T2). p_adj_ = *p*-values corrected for multiple testing, using Bonferroni-Holmes correction. *r* = effect size r (12).

We pooled the FSMC and TAP data by assuming the presence of neurocognitive deficits in the attention domain if two subdomains of the TAP test scored T < 40. Based on this assumption, 54% of PCC patients with severe fatigue were found to have coexisting attentional disorders.

**Figure 2:**
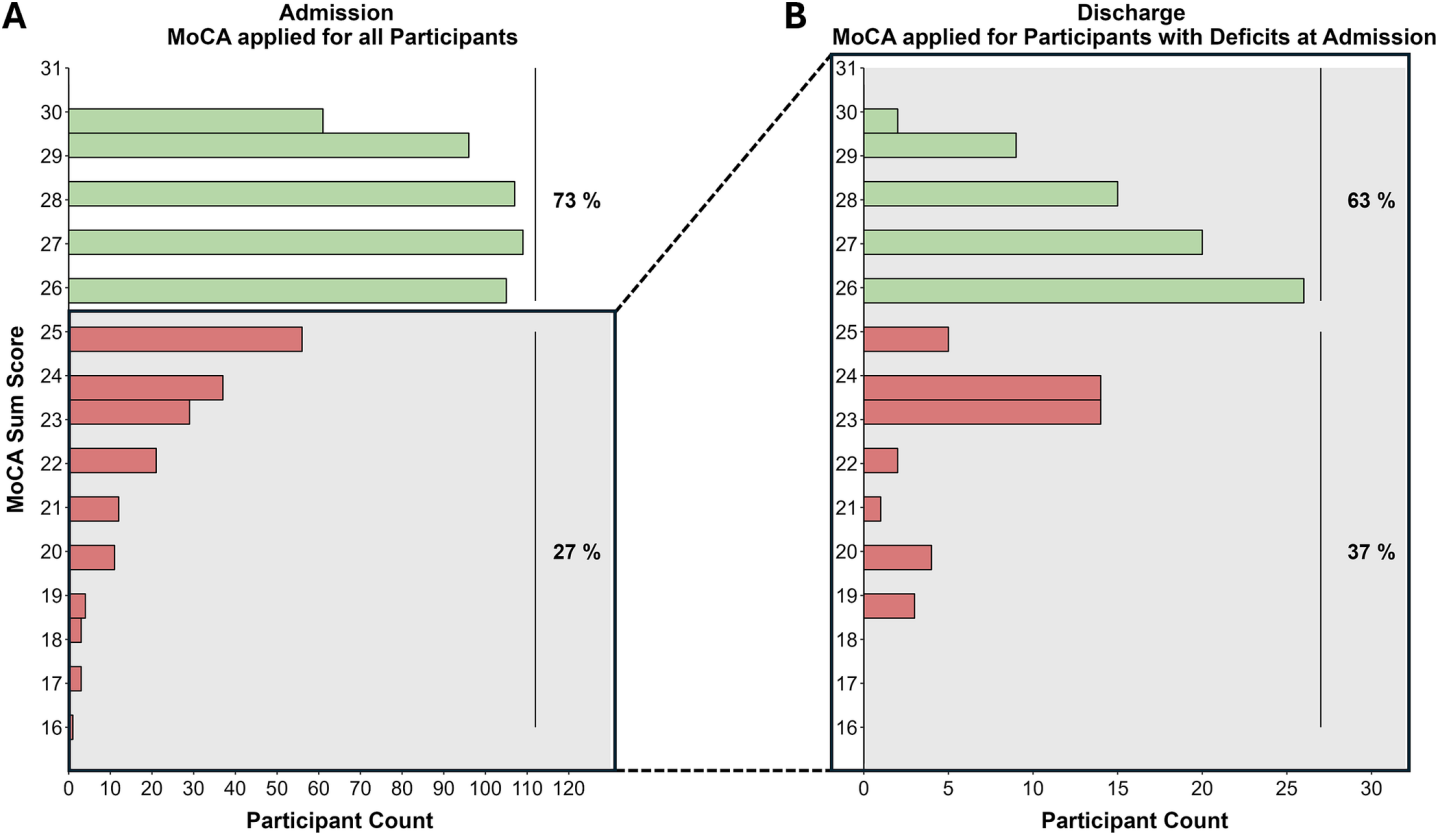
Distribution of MoCA sum scores at admission **(A)** and discharge **(B)**. Green bars indicate normative MoCA scores (≥26), red bars indicate deficits. **(A)** *n_normative_* = 480 (73%); *n_impaired_* = 177 (27%). **(B)** *n_normative_* = 72 (63%); *n_impaired_* = 43 (37%).

Applying the MoCA test at admission showed suspicious results (cut off <26) in 177 out of 657 individuals (27%) of the participants, which normalized in 72 out of 115 (63%) at follow-up on discharge (see Figure 2).

To detect clinically relevant changes in MoCA scores over time, Krishnan et al. (15) calculated a reliable change index (16) of ±1.73 based on a healthy sample. Within our sample, 68 of 115 (59%) impaired patients at T1 (cutoff <26) showed an improvement of at least 2 points on the MoCA score, while 3 of 115 (3%) participants showed a clinically relevant worsening of the MoCA score (≤ −2).

For the evaluation of the specificity and sensitivity of the MoCA to detect neurocognitive deficits in attention domain, we compared the prevalence of abnormal MoCA sum score at T1 with the prevalence of neurocognitive deficits in attention domain at T1. The ladder we defined as the prevalence of deficits as showing impairments in at least two domains of the TAP. It should be noted, that we included only participants with complete MoCA and TAP data at T1 (*N* = 875) for this subanalysis. The MoCA test has a specificity of 83% to predict a deficit in attention, but only a sensitivity of 37%.

## Discussion

With the aim of assessing the prevalence of neuropsychological deficits in PCC and depicting potential changes throughout the course of indoor rehabilitation, we conducted a comprehensive, standardized assessment of attention at the beginning and end of a multidisciplinary rehabilitation program.

Performing subtests of the TAP, we measured a high prevalence of attention deficits, particularly in the domains alertness and sustained attention. Compared to admission, we found significant overall improvement in all subdomains at discharge, especially large effects among those admitted with impairments.

Our data adds to existing knowledge on overall cognitive slowing in post COVID patients (17), indicated by slow reaction times in alertness that were highly prevalent in our sample. Further, we provided evidence that a rehabilitation program can lead to improvements on a group level and clinically relevant individual improvements. On an individual level, we could show that patients with clinically relevant improvements in cognitive slowing were more likely to improve in overall and cognitive fatigue symptoms. While this effect was small, it adds to the findings on the interplay of cognitive slowing and fatigue symptoms.

Analyses on individual levels revealed that while a higher proportion of patients improve within the subtests of attention, some individuals (1.5–11.7%) show worse performance at discharge compared to admission. This proportion being rather small, this finding highlights the importance of individualized diagnostics, treatment plans, and evaluation, and might encourage further research to evaluate interventions on an individual basis in addition to group comparisons. This aligns with publications highlight the heterogeneity of the post COVID population and the need for comprehensive, interdisciplinary diagnostics and individualized treatment. This, however, acquires additional resources, as discussed by, e.g., Hayden (18).

Of the 27% of participants with suspicious scores on the MoCA at admission, 63% improved to a normative level during rehabilitation, also indicating a positive effect of the rehabilitation program in this subgroup. For the interpretation of this effect, it should be considered that we observed baseline differences in MoCA scores, with T2-dropouts showing lower performance than the analyzed complete sample. However, this group difference only had a small effect size (*r* = 0.10), suggesting a small potential bias.

The MoCA test did not prove effective as a screening tool for detecting attention deficits in the participating PCC patients. It was suspicious in only 27% of the cases, and thus only predicted the presence of attentional deficits (measured with the TAP) with a sensitivity of 37%.

Neuropsychological studies have found that the executive functions are particularly severely impaired in PCC patients (19). Others emphasize the importance of attention deficits (20, 21). In addition to the findings of Ariza et al. (22), our data show the importance of conducting a differentiated test of attention.

It should be noted that the same version of the MoCA and TAP was applied at admission and discharge, so that learning effects cannot be ruled out. However, these could be minimal since several weeks lie between the measurement points (23).

Furthermore, assessment was only possible for deficits in the attention domain; other neurological subdomains of the MoCA (e.g., memory) were not extensively explored. Further investigations could explore whether adjusted cut-off values in the MoCA, combined with items from other questionnaires, offer a pragmatic screening alternative.

Fatigue has a very high prevalence and amount in our cohort.

This is interesting because between 6 and 24 months after infection, about half of all fatigue cases resolve in a population-based study (24). Therefore our PCC patient cohort is considered to be a selected population. Our PCC patients were on average 29 months post-infection and can be considered chronic patients. Despite such a high degree of chronicity, a substantial and significant effect of indoor rehabilitation was observed. To our knowledge, this is the first prospective study to demonstrate such effects in a large patient population with PCC.

However, the impact on fatigue symptoms was less pronounced compared to attention deficits. Consequently, the improvement in self-reported cognitive fatigue and measured cognitive performance does not appear to align within our sample. Additionally, our findings revealed that neurocognitive deficits in the attention domain (defined as deficits in at least two subdomains of attention, as measured by the TAP) coexist with severe fatigue (FSMC) in 54% of cases. This suggests a significant overlap between attention deficits and severe fatigue; however, attention deficits alone do not provide a complete explanation for the presence of fatigue in our sample.

Other studies have similarly discovered that self-reported fatigue measures do not always correlate with reaction times (25). This discrepancy may be due to the measurement of two distinct constructs: While the FSMC assesses perceived fatigue symptoms as a stable trait, the TAP measures cognitive performance as a state.

In this context, the question of the influence of psychological, respectively, psychosocial factors naturally arises. Numerous studies show a certain psychological predisposition for the onset of a PCC (26–28). This question was examined in detail in a subset at one of the participating centers of our PoCoRe study, and Kupferschmitt and colleagues were able to demonstrate a significant improvement in the PHQ-9 (a measure of depression) as a result of indoor rehabilitation treatment at one of the participating centers. However, this did not correlate with the measured changes in the neuropsychological assessment (29).

In a highly nuanced combination of neuropsychological tests and the Patient-Health- Questionnaire- 9 to assess depressive symptoms, Morawa and colleagues found frequently deficient neuropsychological parameters. Clinically relevant depressive symptoms were associated with an elevated risk for an impairment regarding some cognitive functions (30).

It should be noted, however, that the participating clinics also addressed the psychological aspects of rehabilitation needs and developed and evaluated their own cognitive behavioral therapy (CBT) procedures for this clientele (31).

Overall, our study shows desired rehabilitation effects, meaning improvement in the tested neuropsychological domains and fatigue at discharge compared to admission. One limitation is that we cannot generalize this statement to severely affected individuals, as our sample consisted of individuals with a sufficiently good overall health status to be eligible for rehabilitation, due to the German healthcare system regulations.

Another limitation of our study is the absence of a control group, which means we cannot ascertain whether the improvements observed are attributable solely to the rehabilitation program or could also be attributed to spontaneous remission. However, since the PCC patients had already experienced considerable chronification, spontaneous remission is unlikely to be the cause of these improvements.

Another limitation, however, is the considerable drop-out rate among participants for neuropsychological testing at the time of discharge. Since there were no significant differences between this group and the group of participants with a complete TAP examination when comparing FSMC values and TAP examination results at T1, we believe that our results are still representative. For the interpretation of the improvements in MoCA-Scores, it should be considered that we observed baseline differences in MoCA scores, with T2-dropouts showing lower performance than the analyzed complete sample. However, this group difference only had a small effect size (*r* = 0.10), suggesting a small potential bias. Still, potential training effects cannot be excluded.

The reasons for discontinuation were not exclusively due to the refusal of the rehabilitants to undergo a second TAP examination, but also were related to other reasons, for example expiration of funding commitments on the part of the payers or personal reasons that made it necessary to discontinue rehabilitation.

In the future, it seems desirable to combine the data collected here with further data on the psychological outcomes of the participants. In addition, the data indicate the need for intensive neuropsychological assessment and therapy as part of rehabilitation. The aftercare situation is particularly worrying as many as 50% of patients are discharged from rehabilitation with persistent neurocognitive deficits in the attention domain and were searching for further intensive neuropsychological training required. A catamnesis of the PoCoRe cohort 6 months after discharge from rehabilitation will show how the rehabilitation procedures and other contextual factors have affected quality of life and social and occupational participation.

## Conclusion

In a large cohort we have shown the high prevalence of neuropsychological deficits and fatigue among PCC patients who generally benefited from indoor rehabilitation in specialized centers. Given the so far unresolved overlap with self-reported fatigue, depressive symptoms, and other factors such as sleep quality, a comprehensive neuropsychological assessment is essential for developing an individualized treatment plan. Screening instruments need to be evaluated carefully for adequate sensitivity. This underscores the need for improved neuropsychological care in both rehabilitation and outpatient settings.

## Data Availability

The datasets presented in this article are not readily available they are available on request and after verification. Requests to access the datasets should be directed to Thilo Hinterberger, Thilo.Hinterberger@ukr.de.
