# Supplementary material for: Neuropsychological outcome of indoor rehabilitation in post-COVID-19 condition—results of the PoCoRe study
Source: Front Neurol. 2025 Jan 6;15:1486751. doi: 10.3389/fneur.2024.1486751 (PMC11743264; doi:10.3389/fneur.2024.1486751)
Supplement: Supplementary file 1 [file Table_1.docx]

Tab. 1 Interventions

| **Clinic** | **Psychoeducation** | **Exercise therapy / physical therapy** | **Relaxation** | **Psychotherapy** | | **Respiratory therapy** | **cognitive training** | **Creative Therapy** | **other** |
| --- | --- | --- | --- | --- | --- | --- | --- | --- | --- |
|  |  |  |  | ***Group*** | ***single session*** |  |  |  |  |
| **Rehab Centre Todtmoos** | |  |  |  |  |  |  |  |  |
| Pneumological departement | 1x per rehab/60 min *Post-COVID Pneumology*  1x per rehab/90 min *Post-COVID psychosomatic perspective*  1x per rehab/60 min  *Introduction to exercise therapy* | 3x per week/30 min exercise therapy  5x per week/30 min monitored ergometer training | 3x per week/30 min relaxation therapy  1x per week/60 min QiGong (as needed) | 2x per rehab/90 min | 1x per rehab/45 min | 4x per week/30 min | as needed (Freshminder) | Ergotherapy  (as needed)  Art therapy  (as needed) | 1x per week/60 min pacing-group  olfactory training (as needed)  nutrition advice  (as needed)  Social service  (as needed) |
| Psychosomatic Department |  | 1x per week/90 min exercise therapy    4x per week/30 min monitored ergometer training | 2x per week/30 min relaxation therapy | 2x per week/90 min  1x per week/60 min | 1x per week/30 min | 2x per week/30 min |  | 1x per week/90 min Ergotherapy  1x per week/90 min Art therapy |  |
| Psycho-pneumological departement |  | 2x per week/60 min exercise therapy  4x per week/30 min monitored ergometer training | 2x per week/30 min relaxation therapy | 3x per week/90 min | 1x per week/60 min | 5x per week/30 min |  | 1x per week/90 min Ergotherapy |  |
| **Reha Centre Seehof** | |  |  |  |  |  |  |  |  |
| Psycho-cardiolocical rehab | 1x per rehab/60 min *Post-COVID*  1x per rehab/30 min  *Introduction to exercise therapy*  Mulltiple times per rehab/60 min  *psychosomatic or cardiological topics*  1x per rehab/45 min  *Introduction cognitive training* | 2x per week/30 min exercise therapy „COfit“  2x per week/30 min monitored ergometer training  2x per week/45 min Nordic Walking (as needed)  2x per week 30 min aqua fitness (as needed) | 2x per week/45 min  Relaxation therapy  1x per week/60 min QiGong  Hydrojet massage (as needed) | 2x per week/90 min | 2x per week/30 min  Or  1x per week/60 min | 2x per week/30 min | 2x per week/60 min Group  3x rehab/30 min  Single session  accompanying training tasks | 1x per week/ 90 min Ergotherapy  Art therapy  (as needed)  Therapeutic dance (as needed) | nutrition advice  (as needed)  Social service  (as needed)  2x per week/ 30 min Mindfulness group  Aroma therapy (as needed) |
| **Gelderland Clinic** | |  |  |  |  |  |  |  |  |
| Psychosomatic rehab | 1x per rehab/60 min *Post-COVID and Naturotherapy*  2x per rehab/60 min *Naturotherapy for stress at work*  1x per rehab/60 min  *Introduction cognitive training*  1x per rehab/30 min  *Introduction cognitive training Freshminder* | 1x per week/45 min monitored strenght training  1x per week/45 min monitored coordination training  Or  1x per week/30 min aqua fitness  1x per week/60 min Walking | 1x per week/45 min QiGong or Yoga  1x per week/45 min meditative moving | 2x per week/ 75 min  1x per week/ 60 min | 1x per week/30 min | 2x per week/30 min  Group  2x per rehab/ 30 min single session | 2x per week/30 min Group | 1x per week/45 min meditative dancing  or  1x per week/45 min meditative forest bathing  1x per week/30 min dew stepping  Ergotherapy (as needed) | nutrition advice  (as needed)  Social service  (as needed)  1x per week/ 30 min Mindfulness group  Aroma therapy (as needed) |
| **Schmieder Clinics** | |  |  |  |  |  |  |  |  |
| Neurological rehab  *Note.* Patients have approx. 19-21 therapy sessions per week in an individual therapy plan | 1x per rehab *Post-COVID*  Multiple times per rehab, different topics like *smoking cessation, dietetics, overweight, muscular deficits* | After performance level assessment:  stamina training  strength training  Vibration platform training | As needed (PMR) | As needed | As needed | breathing therapy group  guided self-exercise training with breathing therapy device  oxygen therapy  (as needed) | Assessment and training as needed | Daily life training  (as needed)  motor-functional training  (as needed) | olfactory training  (as needed)  nutrition advice  (as needed)  Social service  (as needed) |
| **Clinic Westerwald** | |  |  |  |  |  |  |  |  |
| Neurological rehab  *Note.* Patients have a very individual therapy plan | 1x per rehab *Post-COVID*  Multiple times per rehab, different topics like *fatigue, headache, dietetics,*  *Smoking cessation* | After performance level assessment:  stamina training  strength training  Vibration platform training | As needed (PMR, autogenic training) | none | 1x per week/ 60 min (as needed) | As needed | Assessment and training (CogniPlus) as needed | Compensatory and memory strategies (as needed) | olfactory training  (as needed), including Kaiteki maneuver with local application of vitamin A  Speech therapy (as needed)  Social service (as needed |
| *Note.* The rehabilitation concepts of the neurological clinics are so individual that it is not possible to make any useful and schematic statements about therapy times in advance. | | | | | | | | | |
