# Supplementary material for: Neuropsychological outcome of indoor rehabilitation in post-COVID-19 condition—results of the PoCoRe study
Source: Front Neurol. 2025 Jan 6;15:1486751. doi: 10.3389/fneur.2024.1486751 (PMC11743264; doi:10.3389/fneur.2024.1486751)
Supplement: Supplementary file 2 [file Table_2.docx]

Tab. 2 Overview of the assesments used

| **Instruments** | **Domains covered** | **Items** |
| --- | --- | --- |
| ***Psychological Assessment*** |  |  |
| **Regensburg COVID Documentation (ReCoRD).** | primary COVID-19 symptoms, current post-COVID symptoms (25 frequently reported complaints of different organ systems); relevant medical history | 25 |
| **Work Ability Index (WAI).**  [Ilmarinen, 2006] | demands of the job, health status, resources of the worker | 7 |
| **Personal Health Questionnaire (PHQ).**  [Spitzer et al., 1999] | depressive, anxiety,somatoform disorders, psychosocial functionality, stressors, critical life events | 78 |
| **Dissability Assessment (WHO-DAS 2.0).**  [Üstün et al., 2010] | Cognition (understanding & communicating), Mobility (moving & getting around), Self-care (hygiene, dressing, eating & staying alone), Getting along (interacting with other people), Life activities (domestic responsibilities, leisure, work & school) and Participation (joining in community activities) | 36 |
| **Life Skills (LK-18). [**Hinterberger, Walter & Galuska, 2021] | well-being, self-regulation, commitment, sense of purpose, self-efficacy and social contacts | 18 |
| **Fatigue Scale for Motor Functioning and Cognition (FSMC).**  [Penner et al., 2019] | subjectively experienced fatigue symptoms, graduation of cognitive and motor fatigue | 20 |
| **Avoidance-Endurance Questionnaire (AEQ).** [Hasenbring et al., 2009]  (alternatively: Avoidance-Endurance Questionnaire, short Version (AE-FS). ) | fear-avoidance responses and endurance-related responses (cheerful-suppressive way and a distress-endurance way). | 49 |
| ***Neurological Assessment*** |  |  |
| **Montreal Cognitive Assessment (MoCA).** [Nasreddine et al., 2005]. | various cognitive abilities such as memory, language, contextual thinking, attention and concentration, behaviour, arithmetic, temporal and spatial orientation and the ability to recognise complex shapes and patterns | 30 |
| **Test Battery for Attention (TAP).**  [Scherwath et al., 2008]. | Intensity of attention, Executive functions, control of the focus of attention, Attentional selectivity, focused attention, visuo-spatial attention | 45 min |
| ***Somatic Assessment*** |  |  |
| **Six-Minute Walk Test (6MWT).**  [Enright, 2003]. | assess and control cardiovascular and pulmonary performance below the anaerobic threshold |  |
| **Spiroergometry.**  [Kroidl et al., 2014]. | function of the heart, circulation, respiration and muscle metabolism in a relaxed state and under increasing physical stress up to maximum load |  |
| **Pulmonary function test**  [Tomas, 2018] | Functional and performance capacity of the lungs and bronchial tubes; monitoring the course and therapeutic success of lung diseases |  |
| **Blood gas analysis and laboratory (blood test)**  [Haber, 2012; Boemke et al., 2004] | Blood test that measures how much carbon dioxide and oxygen are in the blood; conclusions can be drawn about the health of the heart and lungs |  |
| **Caridac echo**  [Picard & Weiner, 2020; Carmeli et al., 2020] | information about the structure of the heart (e.g. size of the heart chambers, function of the heart valves, thickness of the heart muscle) |  |
| **24-h ECG**  [Mehraeen et al., 2020, Ståhlberg et al., 2021] | Information about how the heart functions under everyday conditions |  |
| ***Post-Exercise Malaise / Adverse events Monitoring*** |  |  |
| **Structured recording of Adverse Event / Fatigue** | fatigue level, post-exercise malaise / adverse events |  |
